# Bis(furan-2-ylcarbon­yl) diselenide

**DOI:** 10.1107/S160053681102085X

**Published:** 2011-06-04

**Authors:** David B. Cordes, Guoxiong Hua, Alexandra M. Z. Slawin, J. Derek Woollins

**Affiliations:** a School of Chemistry, University of St Andrews, St Andrews, Fife KY16 9ST, Scotland

## Abstract

The title mol­ecule, C_10_H_6_O_4_Se_2_, lies on a twofold rotation axis. The Se—Se bond length of 2.305 (3) Å is similar to that in diphenyl diselenide [2.3066 (7) and 2.3073 (10) Å for the *P* and *M* isomers, respectively] and longer than that in 1,8-diseleno­naph­thalene [2.0879 (8) Å]. The mol­ecule adopts a *gauche* conformation with respect to the C=O groups.

## Related literature

For background information and the structure of diphenyl diselenide, see: Fuller *et al.* (2010 ▶). For the structure of 1,8-diselenona­phthalene, see: Aucott *et al.* (2004 ▶).
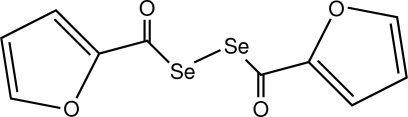


## Experimental

### 

#### Crystal data

C_10_H_6_O_4_Se_2_
*M* *_r_* = 348.07
Orthorhombic, 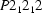

*a* = 9.615 (8) Å
*b* = 14.132 (14) Å
*c* = 3.991 (4) Å
*V* = 542.4 (9) Å^3^
*Z* = 2
Mo *K*α radiation
μ = 6.81 mm^−1^
*T* = 125 K
0.18 × 0.12 × 0.03 mm

#### Data collection

Rigaku Saturn70 diffractometer
Absorption correction: multi-scan (*REQAB*; Rigaku, 1998 ▶) *T*_min_ = 0.384, *T*_max_ = 0.815
1716 measured reflections
895 independent reflections
873 reflections with *F* ^2^ > 2σ(*F* ^2^)
*R*_int_ = 0.057

#### Refinement

*R*[*F* ^2^ > 2σ(*F* ^2^)] = 0.050
*wR*(*F* ^2^) = 0.131
*S* = 1.09
895 reflections
73 parameters
H-atom parameters constrained
Δρ_max_ = 1.63 e Å^−3^
Δρ_min_ = −2.07 e Å^−3^
Absolute structure: Flack (1983 ▶), 322 Friedel pairs
Flack parameter: 0.03 (5)

### 

Data collection: *CrystalClear* (Rigaku, 2009 ▶); cell refinement: *CrystalClear*; data reduction: *CrystalClear*; program(s) used to solve structure: *SHELXS97* (Sheldrick, 2008 ▶); program(s) used to refine structure: *SHELXL97* (Sheldrick, 2008 ▶); molecular graphics: *CrystalStructure* (Rigaku, 2010 ▶); software used to prepare material for publication: *CrystalStructure*.

## Supplementary Material

Crystal structure: contains datablock(s) global, I. DOI: 10.1107/S160053681102085X/lh5259sup1.cif

Structure factors: contains datablock(s) I. DOI: 10.1107/S160053681102085X/lh5259Isup2.hkl

Supplementary material file. DOI: 10.1107/S160053681102085X/lh5259Isup3.cml

Additional supplementary materials:  crystallographic information; 3D view; checkCIF report

## Figures and Tables

**Fig. 1.**
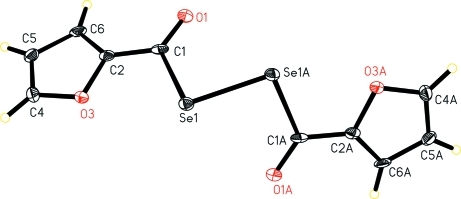
The molecular structure of the title compound with displacement ellipsoids drawn at the 30% probability level (symmetry code: (A) -x, -y+1, z).

## supplementary crystallographic information

### Comment

We have recently reported (Fuller *et al.*, 2010) on the crystallization of Ph-Se-Se-Ph as one isomer. We were interested to see if this homocrystallization occurs for other diselenides. In the title compound, we observe a single isomer in the crystal rather than a mixture of *P* and *M* isomers. The Se—Se bond length of 2.305 (3)Å is similar to that in diphenyldiselenide (2.3066 (7) and 2.3073 (10) Å for *P* and *M* isomers, respectively, Fuller *et al.*, 2010) and longer than that in 1,8-diselenonaphthalene (2.0879 (8)Å, Aucott *et al.*, 2004).

### Experimental

*N-*(furan-2-carbonyl)furan-2-carboxamide (0.205 g, 1.0 mmol) and Woollins reagent (0.54 g, 1.0 mmol) in 20 ml of dry toluene was refluxed for 10 h. Upon cooling to room temperature and removing toluene in vacuum the residue was purified by silica gel column (eluented by 1: 1 hexane / dichloromethane) to give 0.213 g of 1 as a pale yellow solid in 61% yield. Crystals for X-ray data collection were obtained by diffusion of hexane into a dichloromethane solution of (I). ^1^H NMR (CD_2_Cl_2_, δ), 8.01 (d, *J*(H,H) = 8.2 Hz, 2H), 7.54 (d, *J*(H,H) = 8.2 Hz, 2H), 6.90–6.84 (m, 2H) p.p.m.. ^13^C NMR (CD_2_Cl_2_, δ), 179.1 (C=O), 154.1, 148.1, 125.0, 117.1 p.p.m.. ^77^Se NMR (CD_2_Cl_2_, δ), 624.5 p.p.m.. MS (CI^+^, m/*z*), 351 [*M*+H]^+^.

### Refinement

The partial completeness of *ca* 95% data may affect the precision of the structure.  All H atoms were included in calculated positions with C—H = 0.95Å and were refined as riding atoms with *U*_iso_(H) = 1.2 *U*_eq_(C).

### Crystal data

**Table:**

C_10_H_6_O_4_Se_2_	*F*(000) = 332.00
*M**_r_* = 348.07	*D*_x_ = 2.131 Mg m^−^^3^
Orthorhombic, *P*2_1_2_1_2	Mo *K*α radiation, λ = 0.71075 Å
Hall symbol: P 2 2ab	Cell parameters from 1723 reflections
*a* = 9.615 (8) Å	θ = 2.1–26.4°
*b* = 14.132 (14) Å	µ = 6.81 mm^−^^1^
*c* = 3.991 (4) Å	*T* = 125 K
*V* = 542.4 (9) Å^3^	Prism, colourless
*Z* = 2	0.18 × 0.12 × 0.03 mm

### Data collection

**Table:**

Rigaku Saturn70 diffractometer	873 reflections with *F*^2^ > 2σ(*F*^2^)
Detector resolution: 14.629 pixels mm^-1^	*R*_int_ = 0.057
ω scans	θ_max_ = 25.0°
Absorption correction: multi-scan (REQAB; Rigaku, 1998)	*h* = −9→11
*T*_min_ = 0.384, *T*_max_ = 0.815	*k* = −12→16
1716 measured reflections	*l* = −4→4
895 independent reflections	

### Refinement

**Table:**

Refinement on *F*^2^	Hydrogen site location: inferred from neighbouring sites
*R*[*F*^2^ > 2σ(*F*^2^)] = 0.050	H-atom parameters constrained
*wR*(*F*^2^) = 0.131	*w* = 1/[σ^2^(*F*_o_^2^) + (0.0809*P*)^2^ + 2.1662*P*] where *P* = (*F*_o_^2^ + 2*F*_c_^2^)/3
*S* = 1.09	(Δ/σ)_max_ < 0.001
895 reflections	Δρ_max_ = 1.63 e Å^−^^3^
73 parameters	Δρ_min_ = −2.07 e Å^−^^3^
0 restraints	Absolute structure: Flack (1983), 322 Friedel pairs
Primary atom site location: structure-invariant direct methods	Flack parameter: 0.03 (5)
Secondary atom site location: difference Fourier map	

### Special details

**Table:**

Geometry. ENTER SPECIAL DETAILS OF THE MOLECULAR GEOMETRY
Refinement. Refinement was performed using all reflections. The weighted *R*-factor (*wR*) and goodness of fit (*S*) are based on *F*^2^. *R*-factor (gt) are based on *F*. The threshold expression of *F*^2^ > 2.0 σ(*F*^2^) is used only for calculating *R*-factor (gt).

### Fractional atomic coordinates and isotropic or equivalent isotropic displacement parameters (Å^2^)

**Table:**

	*x*	*y*	*z*	*U*_iso_*/*U*_eq_	
Se(1)	−0.03886 (8)	0.42284 (5)	−0.0428 (2)	0.0223 (4)	
O(1)	0.2214 (7)	0.4163 (5)	0.2824 (18)	0.0307 (15)	
O(3)	−0.0133 (6)	0.2267 (4)	0.1408 (17)	0.0245 (14)	
C(1)	0.1221 (8)	0.3698 (6)	0.202 (3)	0.0220 (19)	
C(2)	0.1076 (9)	0.2689 (6)	0.257 (3)	0.0221 (18)	
C(4)	−0.0020 (10)	0.1321 (6)	0.230 (3)	0.029 (3)	
C(5)	0.1174 (9)	0.1177 (6)	0.407 (3)	0.030 (2)	
C(6)	0.1870 (9)	0.2064 (6)	0.420 (3)	0.029 (2)	
H(4)	−0.0677	0.0843	0.1754	0.0354*	
H(5)	0.1481	0.0598	0.5026	0.0358*	
H(6)	0.2737	0.2190	0.5251	0.0346*	

### Atomic displacement parameters (Å^2^)

**Table:**

	*U*^11^	*U*^22^	*U*^33^	*U*^12^	*U*^13^	*U*^23^
Se(1)	0.0186 (5)	0.0255 (5)	0.0228 (5)	−0.0008 (4)	−0.0037 (4)	−0.0008 (4)
O(1)	0.019 (3)	0.029 (3)	0.044 (4)	−0.003 (3)	−0.004 (3)	−0.003 (4)
O(3)	0.017 (3)	0.023 (3)	0.033 (4)	−0.002 (3)	0.000 (3)	0.001 (3)
C(1)	0.011 (4)	0.039 (5)	0.016 (5)	0.002 (4)	−0.002 (4)	−0.006 (4)
C(2)	0.016 (4)	0.031 (4)	0.019 (5)	0.001 (4)	0.006 (4)	−0.008 (4)
C(4)	0.030 (5)	0.021 (4)	0.038 (6)	−0.004 (4)	0.004 (4)	−0.002 (4)
C(5)	0.024 (4)	0.025 (4)	0.041 (6)	0.007 (4)	0.003 (5)	0.006 (5)
C(6)	0.011 (4)	0.039 (5)	0.037 (6)	0.007 (4)	−0.007 (4)	−0.004 (5)

### Geometric parameters (Å, °)

**Table:**

Se(1)—Se(1)^i^	2.305 (3)	C(2)—C(6)	1.337 (13)
Se(1)—C(1)	1.977 (9)	C(4)—C(5)	1.363 (14)
O(1)—C(1)	1.203 (11)	C(5)—C(6)	1.422 (12)
O(3)—C(2)	1.386 (11)	C(4)—H(4)	0.950
O(3)—C(4)	1.388 (10)	C(5)—H(5)	0.950
C(1)—C(2)	1.449 (12)	C(6)—H(6)	0.950
			
Se(1)^i^—Se(1)—C(1)	96.0 (3)	C(4)—C(5)—C(6)	106.5 (8)
C(2)—O(3)—C(4)	105.3 (7)	C(2)—C(6)—C(5)	107.2 (8)
Se(1)—C(1)—O(1)	123.1 (7)	O(3)—C(4)—H(4)	125.009
Se(1)—C(1)—C(2)	111.9 (6)	C(5)—C(4)—H(4)	125.002
O(1)—C(1)—C(2)	125.0 (8)	C(4)—C(5)—H(5)	126.736
O(3)—C(2)—C(1)	117.0 (8)	C(6)—C(5)—H(5)	126.737
O(3)—C(2)—C(6)	110.9 (8)	C(2)—C(6)—H(6)	126.404
C(1)—C(2)—C(6)	132.0 (9)	C(5)—C(6)—H(6)	126.395
O(3)—C(4)—C(5)	110.0 (8)		
			
Se(1)^i^—Se(1)—C(1)—O(1)	−3.6 (7)	Se(1)—C(1)—C(2)—C(6)	−176.7 (7)
Se(1)^i^—Se(1)—C(1)—C(2)	178.9 (5)	O(1)—C(1)—C(2)—O(3)	−178.8 (8)
C(1)—Se(1)—Se(1)^i^—C(1)^i^	−120.5 (3)	O(1)—C(1)—C(2)—C(6)	5.7 (16)
C(2)—O(3)—C(4)—C(5)	2.9 (10)	O(3)—C(2)—C(6)—C(5)	1.3 (11)
C(4)—O(3)—C(2)—C(1)	−178.9 (7)	C(1)—C(2)—C(6)—C(5)	176.9 (9)
C(4)—O(3)—C(2)—C(6)	−2.5 (9)	O(3)—C(4)—C(5)—C(6)	−2.1 (11)
Se(1)—C(1)—C(2)—O(3)	−1.3 (10)	C(4)—C(5)—C(6)—C(2)	0.5 (11)

Symmetry codes: (i) −*x*, −*y*+1, *z*.
